# Green‐brown polymorphism in alpine grasshoppers affects body temperature

**DOI:** 10.1002/ece3.5908

**Published:** 2019-12-08

**Authors:** Günter Köhler, Holger Schielzeth

**Affiliations:** ^1^ Population Ecology Group Institute of Ecology and Evolution Friedrich Schiller University Jena Jena Germany

**Keywords:** Acrididae, body temperature, color polymorphism, color‐mediated thermoregulation, Gomphocerinae, Orthoptera, thermal melanism

## Abstract

1. Ectothermic animals depend on external heat sources for pursuing their daily activities. However, reaching sufficiently high temperature can be limiting at high altitudes, where nights are cold and seasons short. We focus on the role of a green‐brown color polymorphism in grasshoppers from alpine habitats. The green‐brown polymorphism is phylogenetically and spatially widespread among Orthopterans and the eco‐evolutionary processes that contribute to its maintenance have not yet been identified.
2. We here test whether green and brown individuals heat up to different temperatures under field conditions. If they do, this would suggest that thermoregulatory capacity might contribute to the maintenance of the green‐brown polymorphism.
3. We recorded thorax temperatures of individuals sampled and measured under field conditions. Overall, thorax temperatures ranged 1.7–42.1°C. Heat up during morning hours was particularly rapid, and temperatures stabilized between 31 and 36°C during the warm parts of the day. Female body temperatures were significantly higher than body temperatures of males by an average of 2.4°C. We also found that brown morphs were warmer by 1.5°C on average, a pattern that was particularly supported in the polymorphic club‐legged grasshopper *Gomphocerus sibiricus* and the meadow grasshopper *Pseudochorthippus parallelus*.
4. The difference in body temperature between morphs might lead to fitness differences that can contribute to the maintenance of the color polymorphism in combination with other components, such as crypsis, that functionally trade‐off with the ability to heat up. The data may be of more general relevance to the maintenance of a high prevalence polymorphism in Orthopteran insects.

## Graphical abstract

Many grasshopper species occur in green and brown variants within the same local population. The ecological conditions that maintain this shared color polymorphism are not yet understood. We here show that green and brown individuals differ in body temperature under field conditions, suggesting that morph‐specific differences in thermoregulation might contribute to the maintenance of this widespread color polymorphism.

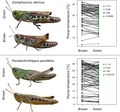

## 1 INTRODUCTION

Ectothermic insects, such as grasshoppers, rely on external heat sources to achieve sufficiently high body temperature for their daily activities, since metabolic heat production is too low to maintain body temperatures independent of ambient temperatures (Chappell & Whitman, 1990; Heinrich, 1993; Uvarov, 1977). The efficiency of thermoregulation in grasshoppers and other ectotherms depends on size, morphology, and reflectance properties of the body and can be modified by microhabitat choice and behavior (Anderson, Tracy, & Abramsky, 1979; Harris, McQuillan, & Hughes, 2015; O'Neill & Rolston, 2007; Parker, 1982; Whitman, 1987). Thermoregulation is an integral part to survival and fitness of ectotherms that influences, for example, the rate of development (Begon, 1983; Coxwell & Bock, 1995), activity patterns (Civantos, Ahnesjö, Forsman, Martin, & Lopez, 2004; Köhler, Samietz, & Wagner, 2001; Whitman, 1987), metabolism (Chappell, 1983), defense against pathogens (Carruthers, Larkin, Firstencel, & Feng, 1992; Elliot, Blanford, & Thomas, 2002; Springate & Thomas, 2005), and rate of reproduction (Samietz & Köhler, 1998).

The color‐mediated thermoregulation hypothesis predicts that body color plays a significant role in the thermal ecology of ectotherms (Geen & Johnston, 2014). A well‐described phenomenon is thermal melanism (Brakefield & Willmer, 1985; Clusella Trullas, van Wyk, & Spotila, 2007; True, 2003) that aims to explain the macroecological gradients of increased frequency of dark individuals under cooler conditions of high latitudes and altitudes (a phenomenon known as Bogert's rule, Bogert, 1949; Gaston et al., 2009). Darker colors typically reflect less and absorb more of the incoming radiation and thus often have a thermoregulatory advantage (Chappell & Whitman, 1990). There is substantial support for the thermal melanism hypothesis, both from comparisons across species (Willmer & Unwin, 1981), but also from studies on within‐species variation (Brakefield & Willmer, 1985; Umbers, Herberstein, & Madin, 2013; Zverev, Kozlov, Forsman, & Zvereva, 2018). Some of the evidence refers to various Orthopterans (e.g., *Melanoplus* sp., Fielding & DeFoliart, 2007; Parsons & Joern, 2014; Pepper & Hastings, 1952; *Tetrix* sp. Forsman, 1997; Forsman, 2011; Forsman, Ringblom, Civantos, & Ahnesjö, 2002; *Phaulacridium* sp., Harris, McQuillan, & Hughes, 2012; Harris et al., 2015). These species, like most Orthopterans, are quite variable in their base color and color patterns and include light‐ and dark‐colored variants that can sometimes be classified into distinct color morphs (Dearn, 1990).

However, no study has yet assessed whether color‐mediated thermoregulation might contribute to the maintenance of a marked green‐brown polymorphism in Orthopterans that is shared across a large number of species (Dearn, 1990; Rowell, 1972). The polymorphism includes many species from both the suborders Ensifera and Caelifera that have been separated about 200 Mya (Misof et al., 2014). About 30% of European Orthopteran species are green‐brown polymorphic (own compilation from field guides) as well as about 45% of the East African Acridid grasshoppers (Rowell, 1972). Indeed, the polymorphism occurs among Orthopterans worldwide and is present in other groups of Polyneoptera, such as in mantises and stick insects. The widespread and apparently stable coexistence of green and brown morphs suggests that the polymorphism is maintained by balancing selection. In some cases, color morph composition varies gradually (e.g., Dearn, 1981; Harris et al., 2012; Köhler, Samietz, & Schielzeth, 2017; Kuyucu & Çağlar, 2016), and in such cases, it is typically the darker brown morph that dominates in cooler habitats (Köhler et al., 2017), thus following Bogert's rule (but see Kuyucu & Çağlar, 2016).

Green and brown morphs differ in the presence or absence of green pigments in the epidermis with the main candidate pigment being biliverdin (Fuzeau‐Braesch, 1972; Shamim, Ranjan, Pandey, & Ramani, 2014). Since brown morphs usually appear darker to the human eye, it has been speculated that they might have a thermoregulatory advantage that potentially trades‐off with other fitness‐related functions (Dieker, Beckmann, Teckentrup, & Schielzeth, 2018; Köhler et al., 2017) such as crypsis and predator avoidance (Ahnesjö & Forsman, 2006; Bond & Kamil, 2006; Pitt, 1999). However, little is known about the thermal biology of green‐brown polymorphic grasshoppers under field conditions (Chappell & Whitman, 1990; Dearn, 1990). Alpine habitats are particularly suitable for detecting thermoregulatory trade‐offs, because temperature and the ability to heat up are limiting at high altitudes (Körner, 2007; Nagy & Grabherr, 2009).

**Figure 1:**
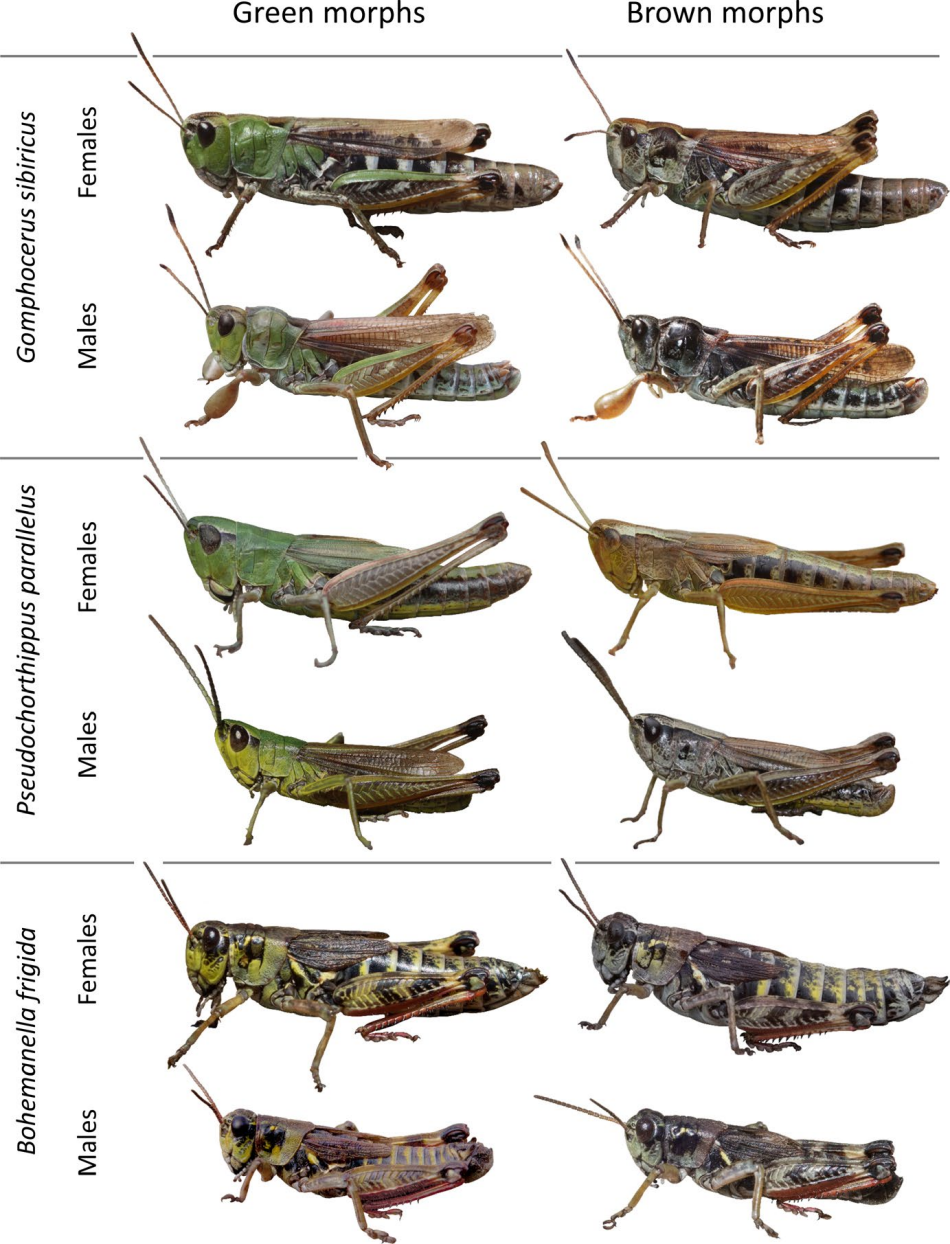
Green and brown color morphs of the color polymorphic club‐legged grasshopper *Gomphocerus sibiricus*, the meadow grasshopper *Pseudochorthippus parallelus* and the high mountain grasshopper *Bohemanella frigida*. Image of green male *Bohemanella* by courtesy of Jürgen Fischer

We here present data on body temperature measurements from grasshoppers field‐caught at 2,000–2,200 m a.s.l. in the Austrian Alps. Specifically, we measured thorax temperature in males and females of 10 species, three of which are distinctly green‐brown polymorphic (Figure 1). The polymorphic species included the club‐legged grasshopper *Gomphocerus sibiricus*, in which green individuals typically represent about 25% of local populations with spatial heterogeneity in morph ratios across the range (Dieker et al., 2018). The club‐legged grasshopper is a high‐altitude species that typically lives above 1,500 m a.s.l. in the European Alps. A second abundant polymorphic species in our analysis is the meadow grasshopper *Pseudochorthippus parallelus*, a more widespread species that occurs at high and low altitudes. Most populations are color polymorphic with a marked altitudinal cline in morph ratios with a sharp increase in brown morphs above 1,500 m a.s.l. (Köhler et al., 2017). The third polymorphic species is the high mountain grasshopper *Bohemanella frigida*, a high‐alpine species that is also sometimes referred to as *Melanoplus frigidus*.

We aimed to test for (a) general daytime trajectories in elevating body, (b) sex differences in body temperatures, and, most importantly, (c) color morph differences in body temperature under highly similar environmental conditions. While there have been a number of studies on thermoregulation in grasshoppers and many other species, including several that address color‐dependent differences, none has yet tested whether the green‐brown polymorphism might affect body temperatures. We find a rapid increase in body temperature during morning hours as well as significant sex and morph differences suggesting a trade‐off that can contribute to the maintenance of color polymorphisms and color morph clines.

## 2 MATERIAL AND METHODS

### 2.1 Sampling

We sampled adult grasshoppers at three locations from the Southern Tauern Mountains near Heiligenblut (Carinthia, Austria), just outside the *Hohe Tauern* National Park. Study plots were located at 2,000–2,220 m a.s.l. on sun‐exposed slopes with alpine vegetation (Table S1). Samples were taken on 4–6 September 1994 on warm, bright, and mostly sunny days, without rain and with little wind. Sampling covered most of the day and ranged between 8:00 a.m. and 17:00 p.m. Grasshoppers were caught with a sweep net, thorax temperature was recorded on spot (i.e., within seconds after capture), and subjects were immediately released after measurements were taken. Individuals of different species, sexes, and color morphs were caught haphazardly, representing the full local community of species. Two teams of two people each were sampling and recording simultaneously.

### 2.2 Measurements

We measured thorax temperature of grasshoppers using the “grab‐and‐stab” technique. After grasshoppers were caught by rapid sweeping, they were fixed within the light gauze net and immediately measured through the meshes with a thermocouple microprobe (Testoterm, 97TE, NiCr‐Ni 0.5 mm). The tip of the probe was rapidly, but gently injected via the thoracal sternum into the thorax. The abdomen was fixed by two fingers through the net so that an influence of the handling person on the thermocouple was avoided. Each measurement lasted for a few seconds, and the constant temperature maintained for about 3 s was registered by a digital recorder (Testoterm 9010) with an accuracy to the nearest 0.1°C. Along with thorax temperature, we recorded species identity, sex, and color morph of each individual as well as time (hh:mm) of measurement.

### 2.3 Morph classification

Green and brown morphs are readily distinguished in three of the 10 species that were recorded (Figure 1). While green morphs show clear, bright green areas, particularly on their thorax and head, brown individuals are characterized by the complete absence of green colors. For *P. parallelus*, we pooled four variants of greenish morphs (green, green‐with‐brown legs, dorsal stipe, and green‐with brown sides, Köhler et al., 2017) into the green class and completely brown individuals into the brown class. For *G. sibiricus*, we pooled two variants of brown morphs (pied and brown, Dieker et al., 2018) into the brown class. In both these species, morph identities are (at least partly) heritable (Köhler, 2006, own unpublished data). Morph classification for *B. frigida* was based on the presence or absence of green colors. The alpine specialist *B. frigida* is sometimes very dark, even in green individuals, and green morphs seem to be rarer in males (as in our sample). Individuals from monomorphic species were classified accordingly by the presence or absence of green. We here present the full dataset, including all 10 species, since these reflect a sample of the full grasshopper species community at the sampling site. The main analysis, however, is focused on the three polymorphic species.

### 2.4 Analysis

We analyzed the data using mixed models and by paired *t* tests on pairs matched for time of catching (see below). The mixed‐model analysis was done combining all species and separately for the three color polymorphic species (that contributed 88% of the data) separated by species. Since the functional relationship of daytime on body temperatures was unknown (and not the prime interest of the study), but sufficient control for temporal heterogeneity is important when testing for color morph effects, we fitted daytime (centered to 12:00) as a linear, quadratic, and cubic term in the linear part of the model and furthermore fitted a random effect *hour of recording* to allow for further temporal heterogeneity. In the analysis that pooled data from all species, we also controlled for species identity as a random effect. The main predictors of interest (sex, morph, and their interaction) were fitted as fixed effects. Binary predictors were centered to zero, such that slopes estimate the difference between groups, but main effects remain interpretable in the presence of interactions (Schielzeth, 2010). Random effects were tested by likelihood ratio tests and fixed effects by *t* tests using the Satterthwaite method for an approximation of degrees of freedom.

Since the mixed‐model analysis hinges on efficient control of temporal heterogeneity and since the functional relationship was not a priori known, we also applied a matched pair analysis that we consider simple and efficient for controlling for temporal heterogeneity. We matched records of the same species and sex, caught at the same location, by the same team and within max. 5 min (median 1 min) of each other. We aimed to maximize the number of pairs while ensuring that no individual appeared in more than one comparison. If multiple potential matches were available within a 5‐min interval, we chose the one closest in time. The matched analysis included 58% of the available observations from the three polymorphic focal species.

These pairs, now post hoc matched for variability in environmental conditions, were analyzed by paired *t* tests. We conducted separate analyses by species and sex. While it is possible to pool the data of the two sexes, a separate analysis can be fruitful as the subsets represent independent data and can act confirmatory if they show similar patterns (see Krause, Krüger, & Schielzeth, 2017 for a justification). A second interest was in sex differences, and for consistency, we proceeded in a similar manner, splitting the data by species and morph and forming matched pairs by sex (effectively using 50% of the observations on the three focal species). All analyses were done in R 3.5.3 (R Core Team, 2019) using the package lme4 version 1.1‐20 (Bates, Mächler, Bolker, & Walker, 2015) for fitting mixed models and the Satterthwaite approximation as implemented in the package lmerTest version 3.0‐1 (Kuznetsova, Brockhoff, & Christensen, 2017).

### 2.5 Analysis history

The data were originally collected in order to describe the temporal trajectory of body temperatures in alpine grasshoppers. Data on color morphs were collected as auxiliary information. With our recent interest in the maintenance of the green‐brown polymorphism, the data were now analyzed with a focus on morph differences. The matching of pairs offers a very efficient way of controlling for variable conditions without further assumptions about temporal and/or spatial heterogeneity. The decision to analyze matched pairs and the matching of pairs was done before testing for color morph differences in other ways. However, the analysis using mixed models offers an obvious alternative and gave very similar results. We present both analyses.

## 3 RESULTS

### 3.1 Thorax temperatures

**Figure 2:**
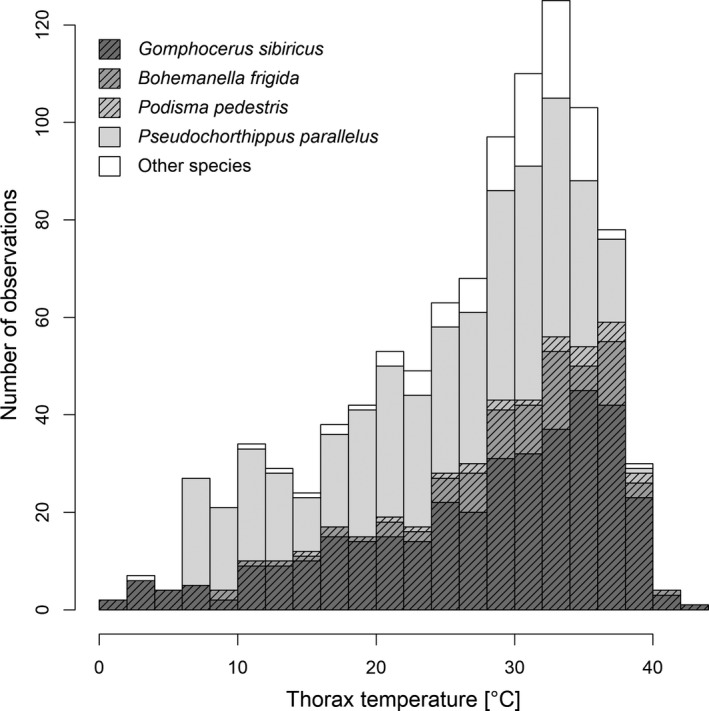
Stacked distribution of thorax temperatures across 1,009 individuals from 10 species of Orthopterans in an alpine environment. Three high‐alpine specialist species are shown with hatched patterns. All other species occur both at high and low altitudes

**Figure 3:**
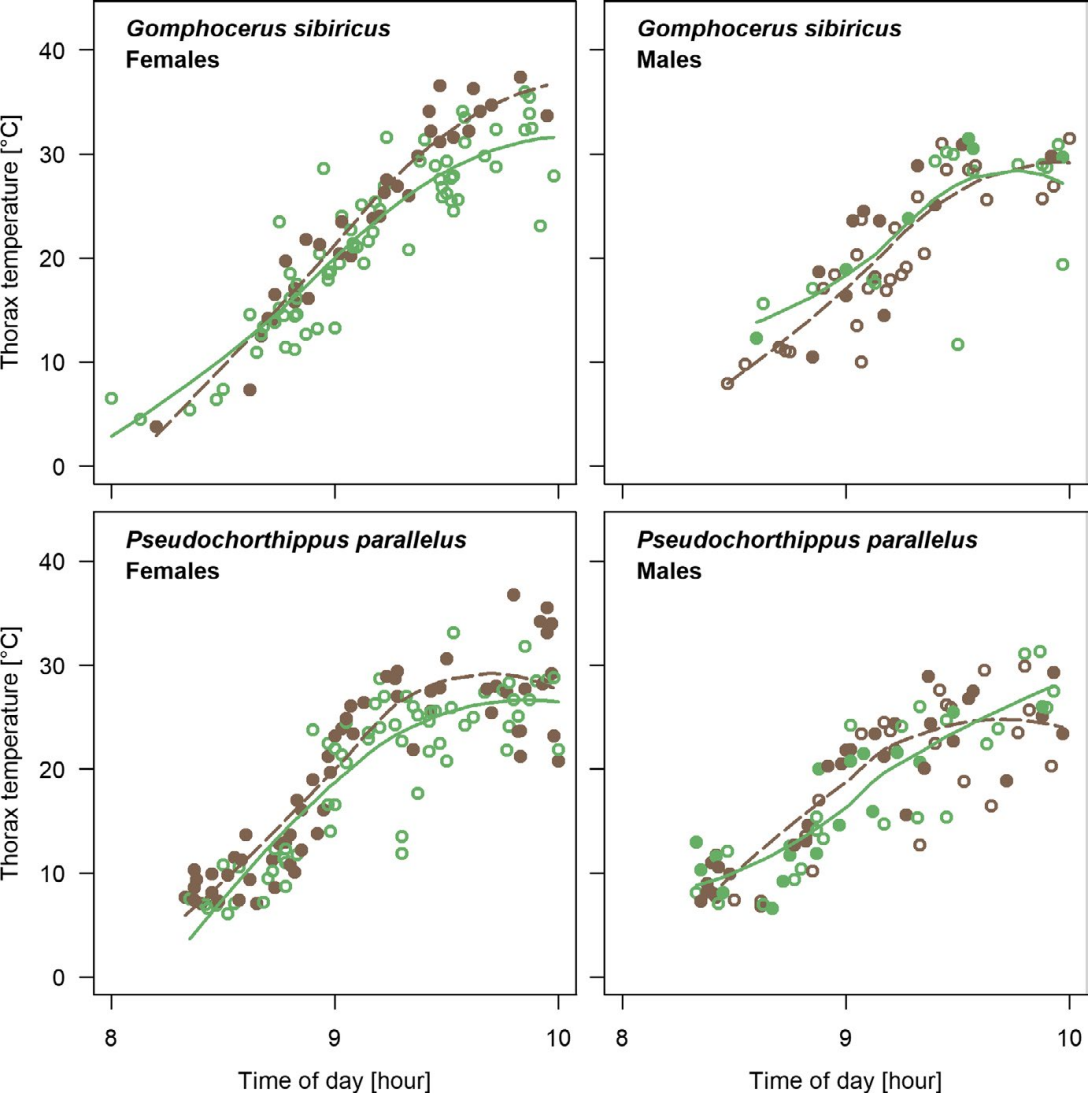
Morning heat up of two green‐brown polymorphic species of grasshoppers as measured by their thorax temperatures. Green morphs are shown with open symbols and brown morphs with filled symbols. Colored lines show loess smoothed trends for brown (dashed lines) and green morphs (solid lines). Figures show only part of the full dataset that was collected in the critical early morning period when individuals heat up

We recorded thorax temperatures of 1,009 field‐caught grasshoppers from 10 species of grasshoppers (Caelifera, Orthoptera). Thorax temperature ranged 1.7–42.1°C, with both highest and lowest values referring to the high‐alpine species *G. sibiricus* (Table 1, Figure 2). Temperatures during the warm periods of the day (10–15 hr) under favorable conditions ranged mostly 31–36°C (interquartile range; Figure S1). The morning heat up phase was characterized by a steep increase in thorax temperatures around 9 a.m. (Figure 3).

**Table 1:** Sample sizes and range of measured thorax temperatures

Species	Total	Female	Male	Brown	Green	*T*_min _[°C]	*T*_max_ [°C]
*Bohemanella frigida* a	84	44	40	23	57	8.7	40.6
*Euthystira brachyptera*	15	8	7	0	15	12.2	35.8
*Gomphocerippus rufus*	47	25	22	47	0	23.8	35.4
*Gomphocerus sibiricus* a	361	231	130	164	185	1.7	42.1
*Omocestus viridulus*	6	4	2	0	6	10.4	32.8
*Podisma pedestris* a	22	13	9	22	0	14.4	39.7
*Pseudochorthippus parallelus*	447	283	164	249	197	6.1	38.7
*Psophus stridulus*	8	4	4	8	0	16	39.6
*Stenobothrus lineatus*	17	9	8	0	17	3.9	36.8
*Stenobothrus rubicundulus*	2	0	2	2	0	33.2	33.2

Note

Morph identities were missing for a small number of individuals.

Abbreviations:* T*_max_ = maximum thorax temperature measured; *T*_min_ = minimal thorax temperature measured.

a High‐alpine specialist species.

### 3.2 Sex differences

Thorax temperatures of females were on average 2.4°C warmer than males (Table 2). Species‐specific analyses using mixed models showed a difference of 3.37 ± 1.03 in *G. sibiricus* (*t*_332.0_ = 3.27, *p* = .0012) and of 2.41 ± 0.64 in *P. parallelus* (*t*_433.7_ = 3.73, *p* = .00021), but no significant differences in *B. frigida* (0.59 ± 2.29, *t*_69.6_ = 0.26, *p* = .80). Matched pair analyses for the two species with most abundant data (*G. sibiricus* and *P. parallelus*) showed significant differences in green as well as in brown morphs of about 1.0–3.5°C (Figure S2).

**Table 2:** Linear mixed‐model analysis of thorax temperatures of 10 species of alpine grasshoppers

Random effects	Variance	Variance ratio	*df*	*χ* ^2^	*p*
Hour of day	13.04	0.47	1	365.89	<10^–15^
Species	1.88	0.07	1	3.85	.0498
Residual	12.66	0.46	1		

Fixed effects	Slope	*SE*	*df*	*t*	*p*
Intercept	37.89	1.29	27.1	29.29	<10^–15^
Time (linear)	2.28	0.49	47.7	4.67	<.0001
Time (squared)	−1.18	0.08	145.5	−14.04	<10^–15^
Time (cubic)	0.08	0.03	407.9	3.27	.00117
Sex = Male	−2.44	0.52	970.2	−4.73	<10^–5^
Morph = Green	−1.52	0.48	972.2	−3.16	.00163
Sex = MaleYN × Morph = Green	0.82	0.48	968.2	1.73	.08478

Note

Time of the day (centered to noontime) was fitted as linear, quadratic, and cubic term to account for nonlinearity in temporal change, and hour of the day was fitted as an additional random effect to account for further deviations from the average trajectory. Sex and morph were binary coded and catered to zero, such that main effects are interpretable in the presence of interactions (Schielzeth, 2010).

### 3.3 Color morph differences

**Figure 4:**
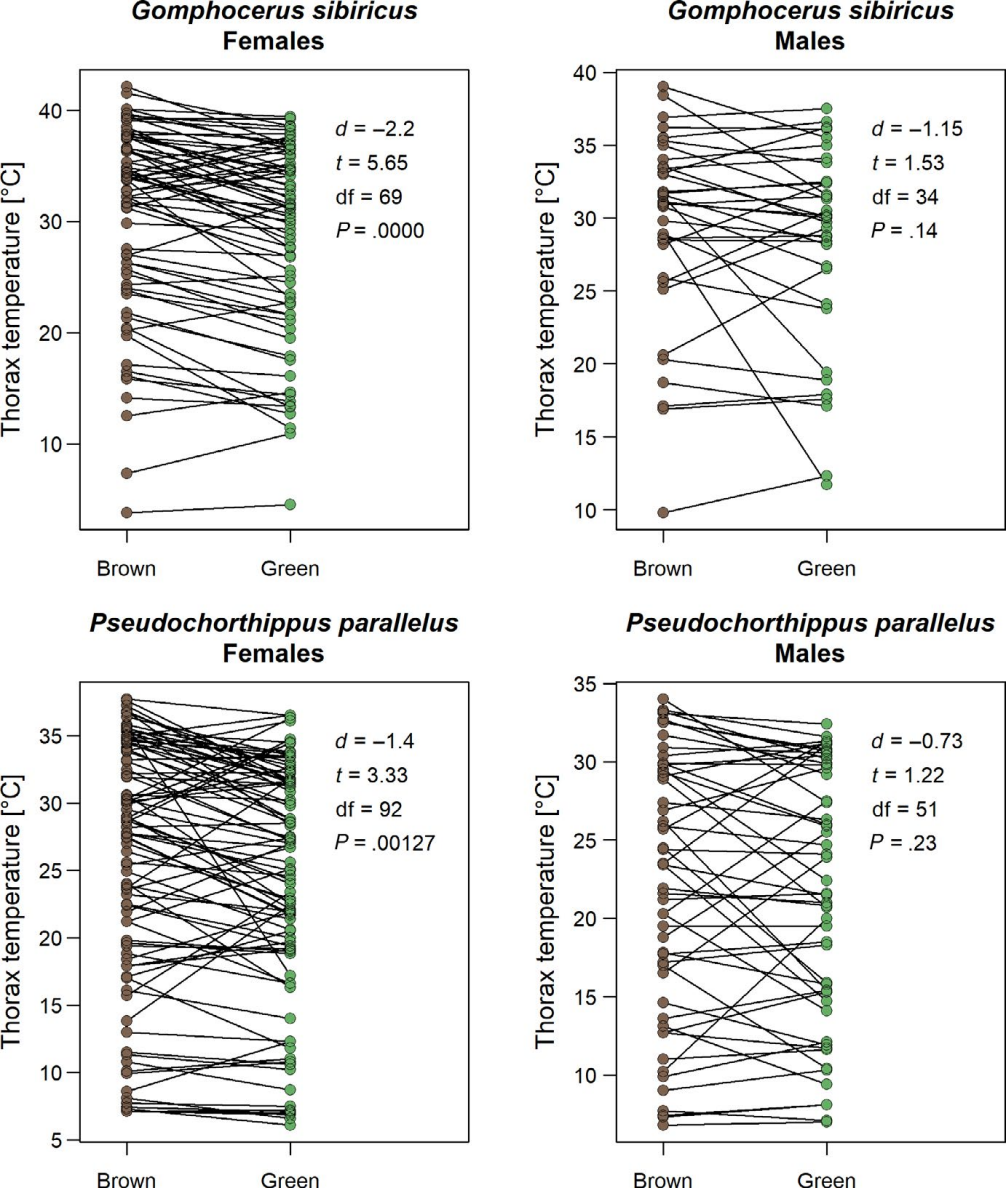
Difference in thorax temperature between matched pairs of brown and green individuals of the same species (*Gomphocerus sibiricus* and *Pseudochorthippus parallelus*, respectively) and sex. The statistics show the result of paired *t* tests (see methods on matched pair analysis)

Thorax temperatures of green morphs were on average −1.5°C cooler than brown morphs (Table 2). Three of the species that we recorded were color polymorphic, and these were the species most abundant in our sample (88% of all records, Figure 2). Species‐specific mixed‐model analyses showed a difference of −1.62 ± 0.92 in *G. sibiricus* (*t*_327.5_ = −1.77, *p* = .077) and of −2.03 ± 0.60 in *P. parallelus* (*t*_434.1_ = −3.38, *p* = .00080), but no significant differences in *B. frigida* (−0.76 ± 2.14, *t*_69.5_ = −0.36, *p* = .72). In none of these cases was there a statistically supported evidence for a sex‐by‐morph interaction (all *p* > .10). Matched pair analyses for *G. sibiricus* (105 matched pairs) and *P. parallelus* (147 matched pairs) that showed that thorax temperatures were lower in green individuals by 1.4–2.2°C in females and 0.7–1.5°C in males (Figure 4). For the more thinly spread *B. frigida*, it was not possible to form a meaningful number of matched pairs.

## 4 DISCUSSION

We here report thorax temperatures in a large sample of 10 alpine grasshopper species. We show that under favorable conditions with weak wind, clear skies, and exposure to sun, thorax temperatures raise rapidly in the mornings and then stabilize around 31–36°C (similar to the range of 32–35°C reported for low‐land populations of *P. parallelus*, Springate & Thomas, 2005). We find that females, which are typically larger than males in Acridid grasshoppers, are on average about 2.4°C warmer than males. In two green‐brown polymorphic species, we find brown individuals to be warmer by about 1.5°C. The third polymorphic species showed a similar, but clearly nonsignificant trend. The species varies substantially in darkness within both morphs, an affect that might dominate morph differences. The data suggest that color‐mediated thermoregulation applies to at least some cases of the green‐brown polymorphism in Orthopterans. Our data refer to realized body temperatures in naturally occurring color variants. The results might thus, in principle, be explained by color‐specific differences in heat absorption, by morph‐specific differences in thermoregulatory behavior (including microhabitat choice), or a combination of both.

Since we used naturally occurring color variants without manipulation of body color, it is not possible to firmly conclude whether body color per se had contributed to the patterns that we observe. Morph‐specific differences in thermoregulatory behavior may have contributed to differences among morphs (Chappell & Whitman, 1990; Samietz, Salser, & Dingle, 2005). So far, there is no indication that morphs differ in thermoregulatory behavior. However, if brown individuals seek out matching dark microhabitats, this may have caused warmer conditions than when green individuals select matching green microhabitats. Matching habitat choice (Edelaar, Siepielski, & Clobert, 2008) may thus potentially contribute to among morph differences in body temperatures. There is no indication that morphs differ in average body size. However, even if behavior or size rather than body color alone contributes to the differences that we observe, this is no less interesting and may affect the conditions for coexistence of different color morphs, including altitudinal clines in color morph composition (Köhler et al., 2017). Our data show that by whatever route, brown individuals are slightly warmer on average and thus have larger activity windows under limited conditions (e.g., on chilly overcast days or days with variable condition). It remains to be seen whether the difference is more or less pronounced under less favorable conditions.

Similar conditions apply to the temperature differences between males and females. Here, we find it somewhat more likely that the sexes differ in thermoregulatory behavior, since males are selected to search for mating partners and to invest in stridulation, two behaviors that may conflict with optimal thermoregulation. In females, egg production depends on temperature and it is therefore possible that females invest more into heating up and maintaining higher body temperature. At the same time, females are significantly larger in all species considered here and thus provide a larger surface for heat absorption and larger volume that may buffer against heat loss. Larger individuals are generally slower to heat up, but achieve greater temperature excess in ectothermic insects (Chown & Nicolson, 2004; Nielsen & Papaj, 2015; Woods, 2013).

A temperature difference of about 1.5°C among color morphs and 2.4°C among sexes may not seem excessively high. Similar magnitudes of color morph differences in body temperature have been reported in color polymorphic garter snakes and *Tetrix* groundhoppers (Bittner, King, & Kerfin, 2002; Forsman, 1995). Such rather small differences may be of significance, in particular during periods of limited ambient conditions during mornings, evenings, on overcast days or late in the season and may affect the activity window and/or pathogen resistance (Springate & Thomas, 2005). Even if differences are less pronounced under less favorable conditions, temperature differences under favorable conditions as reported here will result in longer activity phases for as long as initial temperature differences can be maintained. Even if grasshoppers heat up substantially above ambient temperatures by basking, the time spent basking limits investment in other activities. In particular under conditions of scramble competition for mating partners, even slightly larger activity windows may results in significant differences in fertilization success in males (Punzalan, Rodd, & Rowe, 2008; Zverev et al., 2018). Similarly, because heat accelerates egg development, this may influence fecundity (Samietz & Köhler, 1998; Springate & Thomas, 2005).

Overall, our data give support for the suggestion that the color‐mediated thermoregulation hypothesis applies to the widespread green‐brown polymorphism of grasshoppers. This has previously been untested. Thermoregulation is thus a potentially important component in the eco‐evolutionary dynamics that allows the coexistence of green and brown morphs in local populations.

## CONFLICT OF INTEREST

None declared.

## AUTHOR CONTRIBUTIONS

GK conceived and organized the research stay and (together with group members) the sampling design in the field, contributed to data collection and compilation. HS performed the analysis. GK and HS jointly discussed and interpreted the results. HS drafted the manuscript, and both authors contributed to manuscript revision.

## Data Availability

Data will be deposited on Datadryad (http://www.datadryad.org) upon acceptance for publication.
