# Supplementary material for: Green‐brown polymorphism in alpine grasshoppers affects body temperature
Source: Ecol Evol. 2019 Dec 8;10(1):441–50. doi: 10.1002/ece3.5908 (PMC6972831; doi:10.1002/ece3.5908)
Supplement: Supplementary file 1 [file ECE3-10-441-s001.docx]

Supplementary materials
Green-brown polymorphism in alpine grasshoppers
affects body temperature

Günter Köhler & Holger Schielzeth

**Overview**

**Table S1.** Characteristics of the three study sites.

**Figure S1.** Thorax temperature across the time of the day in six species of grasshoppers.

**Figure S2.** Matched pair analysis of thorax temperature for sex differences.

**Table S1:** Characteristics of the three study sites. Sun exposure was measured by a horizontoscope. Wind speed was measured 1.5 m above ground at the period of field sampling using a using a hand-held cup anemometer.

|  | **Fallbichl** | **Tauerneck** | **Schöneck** |
| --- | --- | --- | --- |
| Latitude/Longitude | 47°04´05´´ N  12°50´33´´ E | 47°03´51´´ N  12°49´45´´ E | 47°03´24´´ N  12°48´09´´ E |
| Altitude | 2.220 m a.s.l. | 2.080 m a.s.l. | 2.000 m a.s.l. |
| Exposition | 29-31 S | 39-42 SW | 24-27 SSE |
| Inclination | 26-32° | 30-34° | 36-39° |
| Vegetation type | Curvulo-Nardetum  (cattle pasture)  dry to wet, ruderal | Aveno-Nardetum, with *Vaccinium* and *Rhododendron* shrubs and patches of *Agrostis* | Seslerio-Semperviretum,  Festucetum, Laserpitio-Calamagrostitetum |
| Vegetation height | <0.10-0.80 m | 0.20-1.00 m | max. 0.80 m |
| Vegetation cover | 50-100% | 100% | 0-100% (with open, stony patches) |
| Sun exposure in August | 5:30 am – 18:15 pm | 5:15 am – 17:00 pm | 7:15 am – 17:30 pm |
| Sun exposure in September | 7:30 am – 17:45 pm | 6:00 am – 16:45 pm | 7:30 am – 17:30 pm |
| Windspeed (m/s) | 0.0 - 1.6 | 0.2 | 0.0-1.0 |
|  |  |  |  |

**Figure S1.** Thorax temperature across the time of the day in six species of grasshoppers. Females are shown as blue triangles and males as orange circles. Lines show loess fits (solid for females, dashed for males).

**Figure S2.** Matched pair analysis of thorax temperature of sex differences. Females and males were match separately by species and color morph. The statistics show the result of paired *t* tests (see methods on matched pair analysis).
